# Cooperation Between Cancer and Fibroblasts in Vascular Mimicry and N2-Type Neutrophil Recruitment *via* Notch2–Jagged1 Interaction in Lung Cancer

**DOI:** 10.3389/fonc.2021.696931

**Published:** 2021-08-17

**Authors:** Ying-Ming Tsai, Kuan-Li Wu, Yu-Wei Liu, Wei-An Chang, Yung-Chi Huang, Chao-Yuan Chang, Pei-Hsun Tsai, Szi-Hui Liao, Jen-Yu Hung, Ya-Ling Hsu

**Affiliations:** ^1^Graduate Institute of Medicine, College of Medicine, Kaohsiung Medical University, Kaohsiung, Taiwan; ^2^Division of Pulmonary and Critical Care Medicine, Kaohsiung Medical University Hospital, Kaohsiung, Taiwan; ^3^School of Medicine, College of Medicine, Kaohsiung Medical University, Kaohsiung, Taiwan; ^4^Drug Development and Value Creation Research Center, Kaohsiung Medical University, Kaohsiung, Taiwan; ^5^Division of Thoracic Surgery, Department of Surgery, Kaohsiung Medical University Hospital, Kaohsiung Medical University, Kaohsiung, Taiwan; ^6^Department of Anatomy, Kaohsiung Medical University, Kaohsiung, Taiwan

**Keywords:** cancer-associated fibroblasts, Jagged1, lung cancer, Notch2, vascular mimicry

## Abstract

**Background:**

Angiogenesis is required for tumor development and metastasis, which is a major part in a pro-tumor microenvironment. Vascular mimicry (VM) is a process in which cancer cells, rather than endothelia, create an alternative perfusion system to support the tumor progression.

**Objectives:**

To validate the role of VM and to develop a strategy to inhibit angiogenesis in lung cancer.

**Methods:**

In this study, we utilized lung cancer samples to verify the existence of VM and conducted several experimental methods to elucidate the molecular pathways.

**Results:**

H1299 and CL1-0 lung cancer cells were unable to form capillary-like structures. VM formation was induced by cancer-associated fibroblast (CAFs) in both *in vitro* and *in vivo* experiments. Notch2–Jagged1 cell–cell contact between cancer cells and CAFs contributes to the formation of VM networks, supported by Notch intracellular domain (NICD) 2 nuclear translocation and N2ICD target gene upregulated in lung cancer cells mixed with CAFs. The polarization of tumor-promoting N2-type neutrophil was increased by VM networks consisting of CAF and cancer cells. The intravasation of cancer cells and N2-type neutrophils were increased because of the loose junctions of VM. Disruption of cancer cell–CAF connections by a γ‐secretase inhibitor enforced the anticancer effect of anti‐vascular endothelial growth factor antibodies in a mouse model.

**Conclusion:**

This study provides the first evidence that CAFs induce lung cancer to create vascular-like networks. These findings suggest a therapeutic opportunity for improving antiangiogenesis therapy in lung cancer.

## Introduction

Lung cancer is the leading cause of cancer-related mortality globally with poorer prognosis compared with that of breast, prostate, and colorectal cancer (1). Although tumor angiogenesis inhibition is a therapeutic target, it remains non-beneficial in lung cancer survival because of redundant proangiogenic pathways or acquired resistance (2). Vascular mimicry (VM) is a process in which tumor cells form vascular-like networks that are not lined with endothelial cells (ECs). These networks contribute to tumor blood supply, which fosters cancer progression, metastasis, and chemoresistance to angiostatic compounds (3, 4). The presence of VM networks in cancer is strongly correlated with poor prognosis (5), which has received attention to develop agents targeting VM. In addition, cancer tissues with VM structures have low sensitivity to angiogenesis inhibitors and angiostatic treatment (6, 7). A growing body of evidence has demonstrated that the interaction between the tumor microenvironment (TME) and tumor cells plays a critical role in cancer development and progression (8, 9). Tumor VM formation with its non-cancer cells as macrophages and fibroblasts is associated with cancer cells invasiveness and metastasis (10, 11) and the interaction of the TME in VM development is not yet understood. Therefore, elucidating mechanisms through which this interaction promotes VM formation is pivotal because they can provide new therapeutic opportunities.

The Notch pathway is a well-ordered, evolutionary conserved pathway and crucial to the regulation of morphological development in multicellular organisms that governs connections between interacting cells (12, 13). Dysregulation in Notch signaling transduction is related to various genetic diseases, including cancers (14, 15). Four Notch receptors (NOTCH1–4) and five ligands (Delta-like proteins [DLL] 1, 3, 4, and Jagged1 and 2) have been identified in the cell membrane surface of mammalian cells (16, 17). The Notch juxtacrine pathway initiates the binding of a Notch ligand on the “signal-sending cell” to the Notch receptor on the “signal-receiving cell.” This interaction of nearby cells triggers two sequential steps of proteolytic cleavage by a disintegrin, metalloproteases 10 and 17 (ADAM10 and ADAM17), and γ-secretase, resulting in the production of the active form of Notch (NICD). The nuclear translocation of NICD promotes the transcriptional activation of multiple Notch downstream target genes, including HES and HEY gene families (12). Notch-mediated cell–cell contact regulates many aspects of cancer biology, such as cancer stemness, angiogenesis, the immune infiltrate, and chemoresistance (18, 19). Studies investigating the role of Notch signaling in the TME have indicated that Notch are involved in both procancer and anticancer effects in different populations in intratumoral heterogeneity (20, 21). Therefore, continuing to identify the precise role of Notch-mediated signaling in forming the TME is essential.

A growing body of evidence has indicated that cancer-associated fibroblasts (CAFs), which are the main cancer component of the TME, play a crucial role in tumor progression in various types of cancer. This study investigated the role of CAFs in VM formation. We concluded that CAFs induce lung cancer to form capillary-like structures, which may provide an intravasation or extravasation channel for cancer cells and tumor-promoting neutrophils, and alter the cancer ability to form VM networks by direct Notch2–Jagged1-mediated contact. Our study provides new insights into understanding the unique function of CAFs crosstalk with cancer cells that accounts for immunomodulation and resistance to antiangiogenic therapy in the TME.

## Materials and Methods

### Lung Cancer Cell Line and CAFs

HUVECs, normal human lung fibroblasts (NHLFs), and human lung cancer cells H1299 were obtained from the American Type Culture Collection (ATCC) and human lung adenocarcinoma CL1-0 cell line was generously provided by Dr. Pan-Chyr Yang (Department of Internal Medicine, National Taiwan University Hospital). H1299 and CL1-0 cells were cultured in RPMI 1640 supplemented with 10% FBS and 1% penicillin–streptomycin. HUVECs were cultured in complete EGM2 medium (Lonza). NHLFs were cultured in fibroblast basal medium supplemented with all components of Fibroblast Growth Kit-Low serum (ATCC). For CAFs generation, lung cancer cells (H1299 and CL1-0) and NHLFs (1.5 × 10^5^) were seeded in transwell inserts (pore size: 1 μm) and a 6-well plate then cultured for 24 h. Mycoplasma was tested using mycoplasma test kits (Mycoalert Mycoplasma Detection Kit, Lonza) every 3 months and H1299 cells were authenticated by short tandem repeat analysis with the Geneprint 10 system kit (B9510, Promega, Madison, WI, USA).

### Immunohistochemistry

Lung tumor samples were obtained from 10 patients with lung cancer admitted to the Division of Pulmonary and Critical Care Medicine at Kaohsiung Medical University Hospital (KMUH), Kaohsiung, Taiwan. The Institutional Review Board of KMUH approved the study’s protocol, and all participants provided written informed consent in accordance with the Declaration of Helsinki. Standard immunohistochemistry (IHC) was performed on paraffin‐embedded tumor tissue sections for CD34 staining (1:100, Catalog #ab81289). After a 0.5% periodic acid-Schiff (PAS) reaction, these sections were treated with Schiff’s solution, kept away from light for 15 min, and then counterstained with hematoxylin. VM was defined as PAS+ and CD34− vascular‐like channels. The results were analyzed through a microscope.

### Tube Formation Assay

Corning^®^ Matrigel^®^ Growth Factor Reduced (GFR) Basement Membrane Matrix, Phenol Red-free, LDEV-free (Corning, Arizona, USA) at 200 μl was plated to 48-well plates at a horizontal level at 4°C overnight that allows the Matrigel to remove air bubbles and distribute evenly. CAFs (CL1-0- and H1299-CAF, 2 x10^4^ cells/each), CL1-0 (4 x10^4^ cells), H1299 (4 x10^4^ cells) cells were stained using PHK26 and PKH67 dye and the re-suspended in serum-free RPMI 1640. Martigel was solidify by incubating at 37°C for 30 min, and CAFs, H1299 or CL1-0 cells, or CAFs mixed with H1299 cells were plated on the top of Martigel with or without various inhibitors [DMSO (0.1%), DAPT (2 μM), or 2.5 mM EGTA]. After 6 or 12 h incubation, the results were analyzed through a fluorescence microscope with 4x phase contrast (Nikon Instruments, Tokyo, Japan). Tubular node and branch points from 4 random fields were counted and averaged. Data were presented as the number of tubular nodes or branch points/field.

### Immunoblot/PLA

Total cell lysate and nuclear fraction were obtained using radioimmunoprecipitation assay (RIPA) lysis buffer (Millipore Corporation, Billerica, MA, USA) and Nuclear Extraction Kit (Active Motif, Carlsbad, CA, USA), respectively. Equal amounts of protein were separated on sodium dodecyl sulfate–polyacrylamide gels. Antibodies against NOTCH1 (catalog no. 3608), NOTCH2 (catalog no. 5732), NOTCH3 (catalog no. 5276), NOTCH4 (catalog no. 2423), Jagged1 (catalog no. 2620), Jagged2 (catalog no. 2210), DLL1 (catalog no. 2588), DLL2 (catalog no. 2483), DLL3 (catalog no. 2589), β-tubulin (catalog no. 2144), vinculin (catalog no. 13901) and glyceraldehyde 3-phosphate dehydrogenase (GAPDH, catalog no. 5174) were obtained from Cell Signaling Technology (Beverly, MA, USA). N2ICD (catalog no. AF3735) was obtained from R&D system. The density of Immunoblot bands was measured using AlphaImager software (Alpha Innotech, San Leandro, CA, USA) software.

Proximity Ligation Assay (PLA) can evaluate protein-protein interactions *in situ* using primary antibodies and then detected with a specially designed pair of oligonucleotide-conjugated secondary antibodies, which can be used to produce a signal only when the two probes have bound in close proximity to each other. PLA was performed using DuoLink^®^ kit (Sigma-Aldrich) according to the manufacturer’s instructions. After blocking, cells were incubated with goat anti-Jagged1 ([1:200], catalog no. PA5-46970, Thermo Fisher Scientific) and rabbit anti-Notch2 ([1:200], Cell signaling technology Inc.) antibodies overnight at 4°C. PLA plus and minus probes for goat and rabbits were added and incubated for 1 h at 37°C, and then ligation–ligase solution was added and incubated for another 0.5 h. Amplification–polymerase solution was subsequently added and incubated for 100 min at 37°C. The slides were mounted with mounting media (Vector Lab., Inc., Burlingame, CA, USA), and the results were analyzed through LSM 700 confocal laser scanning microscopy (Zeiss, Jena, Germany).

### RNA Isolation and Quantitative Reverse Transcription Polymerase Chain Reaction Assay

Total RNA from cells was isolated using TRIzol reagent (Life Technologies). cDNA was prepared using an oligo (dT) primer and reverse transcriptase (Takara, Shiga, Japan) following standard protocols. The levels of mRNA transcripts were measured using real-time analysis with SYBR Green on a StepOnePlus machine (Applied Biosystems, Foster City, CA, United States). The relative mRNA expression levels in cells were normalized to GAPDH. The primers used are listed in **Supplementary Table 1**.

### siRNA Transfection

CAFs or H1299 cells were transfected with ON-TARGET plus SMARTpool human Jagged1, Notch2, ICAM2, or control siRNA (DHARMACON) by using Lipofectamine RNAiMAX transfection reagent. After 48 h of transfection, the knockdown efficiency of siRNA was determined with immunoblotting.

### Neutrophil Generation and Cytokine and Soluble Factor Analysis

All blood samples were collected from patients admitted to the Division of Pulmonary and Critical Care Medicine at KMUH, Kaohsiung, Taiwan. All participants provided written informed consent in accordance with the Declaration of Helsinki. Neutrophils were isolated by MACS technology using an anti-CD66abce MicroBead Kit after the red blood cell (RBC) lysis of human peripheral blood. (Miltenyi Biotec, Cologne, Germany). Neutrophil differentiation was performed to obtain N1 and N2 neutrophils, which were cultured in RPMI 1640 (Lonza, Basel, Switzerland) supplemented with the supernatants of H1299 cells, CAFs, or H1229/CAFs for 6 h. The supernatants of CAFs, CAFs/H1299 cells, H1299 cells, and neutrophils were collected, centrifuged for 10 min at 3,000 g, and stored at −80°C. Various cytokine levels were determined using Luminex Assays (R&D Systems).

### Transmembrane Migration, Permeability, and Cell Adhesion

For transmembrane migration analysis, HUVECs (1 × 10^5^ cells/well) or CAFs mixed with H1299 cells were seeded onto inserts with polyester membranes 8 μm in pore size (EMD Millipore) for 2 days, then H1299 or neutrophil cells were seeded onto inserts for 24 h. The permeability assay was performed using the *In Vitro* Vascular Permeability Assay kit (EMD Millipore). HUVECs, H1299 cells, CAFs, or CAFs mixed with H1299 cells were seeded in a 24-well plate for 2 days. Fluorescein isothiocyanate (FITC)-labeled dextran was added to the top of the cell monolayer for 2 h, then FITC-dextran across the cell monolayer to the bottom of the wells was measured by relative fluorescence excitation at 485 nm and emission at 530 nm using a fluorescence plate reader. For cell adhesion analysis, PKH26-labeled H1299 cells or neutrophils were seeded onto cell monolayers for 30 min. After washing with PBS, adherence of H1299 cells or neutrophils was made visible by using through a fluorescence microscope (Nikon Instruments, Tokyo, Japan).

### Flow Cytometry

To analyze the arginase levels of neutrophils, cells were stained with primary antibodies against arginase or corresponding isotype control antibodies (R&D Systems) after permeabilization. Flow cytometry data were acquired using an Accuri C6 flow cytometer (BD Biosciences) and analyzed using CellQuest (BD Biosciences).

### Animal Model

Equal cell numbers of CAFs, H1299, and CAF/H1299 cells were mixed with ice-cold Matrigel (200 µL, BD Biosciences, San Jose, CA). The mixed solution was subcutaneously injected into nude mice (male, 8 weeks old, BALB/c, n = 6). After 13 days, the Matrigel plugs were harvested and double stained using CD34 antibodies and PAS. All animal care was in accordance with institutional guidelines.

The reinforcement effect of Notch inhibition was assessed using the oral γ-secretase inhibitor DAPT. Each nude mouse (male, 8 weeks old, BALB/c, n = 6) was injected subcutaneously (sc.) in the right flank with CAF/H1299 cells (1 × 106) mixed with Matrigel (3:1, BD Biosciences). After palpable tumors developed (average volume, 50 mm3), mice were randomized to receive different treatments. Mice received immunoglobulin G (IgG, IP, 10 μg/mouse, 2 times/week, n=6) or anti-VEGF antibodies (IP, 10 μg/mouse, 2 times/week, n=6) with or without DAPT (po, 5 mg/kg, 3 times/week, 4 weeks, n=6). Tumor growth was monitored for 4 weeks from the first treatment. Tumor volume (V) was measured every day with calipers and calculated according to the formula V= 0.5W2L (W = tumor width; L = tumor length). All animal experiments were performed in accordance with approval of the Institutional Animal Care and Use Committee of Kaohsiung Medical University.

### Statistical Analyses

Data are expressed as the mean ± standard deviation. Two treatment groups were compared using Student’s t test. Multiple group comparisons were performed using two-way analysis of variance with Tukey’s *post hoc* test. Correlations between groups were analyzed using Pearson’s correlation coefficient. GraphPad Prism version 7.04 (GraphPad Inc., La Jolla, CA) was used for statistical analyses. Results were considered statistically significant when P < 0.05.

## Results

### CAFs Increase VM Formation in Lung Cancer

**Figure 1:**
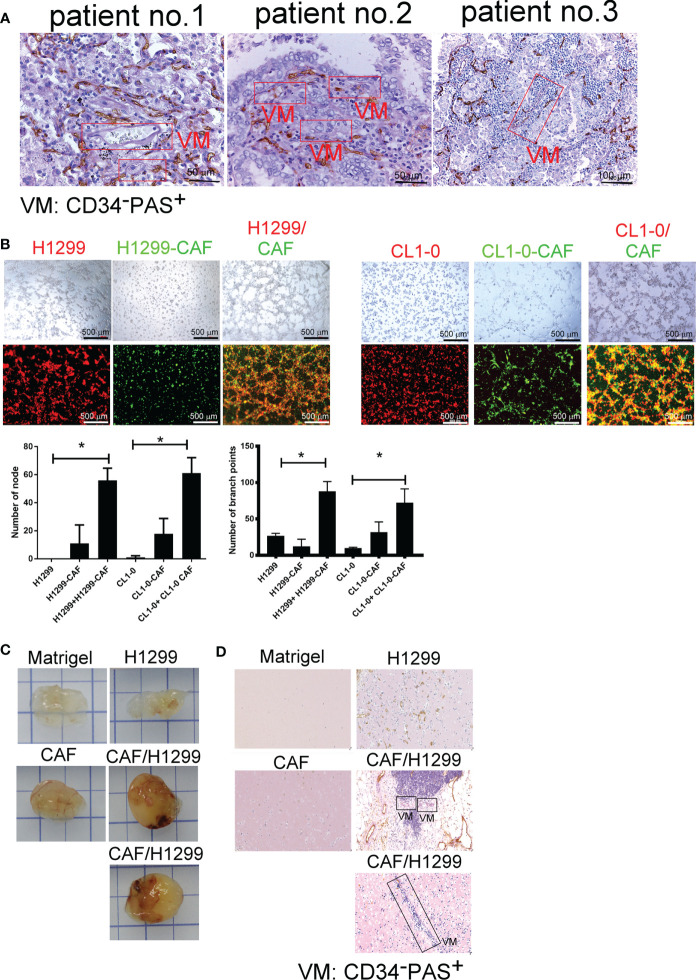
Cancer-associated fibroblasts (CAFs) promote vascular mimicry (VM) in lung cancer. **(A)** VM in tumor of patients with lung cancer. The lung tumor sections were stained with CD34 and PAS. PAS+ and CD34− vascular-like channels were defined as VM. CAFs promoted VM **(B)** *in vitro* (upper: bright-field) and **(C)** *in vivo*. **(D)** The IHC staining of tumor section of mice. H1299 cells (PKH26 staining, red), CL1-0 (PKH26 staining, red), their CAFs (PKH67 staining, green), or lung cancer cells (H1299 or CL1-0) mixed with CAFs and then seeded on the top of growth factor reduced Matrigel for 6 (h) The VM structure was observed using a fluorescence microscope *in vitro*. Matrigel plus VM structure as presented by immunohistochemical (PAS, CD34) staining. H1299, CL1-0, CAFs, or lung cancer cells plus CAFs were mixed with Matrigel. Matrigel plugs were harvested 13 days after subcutaneous injection into nude mice. The presence of VM network was determined through PAS and CD34 staining. VM, vascular mimicry. Each value is the mean ± SD of three determinations; *p < 0.05.

To gain general insights on the clinical association of VM with lung cancer, CD34 and periodic acid-Schiff (PAS) double staining were used to identify VM (22) in 10 lung cancer samples. As illustrated in **Figure 1A**, the structure of VM (defined as PAS+, CD34−) was observed in tumor tissue of patients with lung cancer (3/10) and the characteristics were listed in **Table 1**. The three cases of tumor VM formation were in stage II-IV. In the cell model, we generated CAFs by coculturing normal human lung fibroblast with cancer cells for 24 h, and identified α-smooth muscle actin (α-SMA) (23, 24) was increased in fibroblast when they were stimulated by cancer cells (**Supplementary Figure 1A**). We used lung cancer H1299 and CL1-0 cells to assess the VM of cancer. Our results showed that the cell density and time affected the ability of H1299 on VM formation (**Supplementary Figure 1B**), we thus assessed the effect of CAFs by seeding H1299 at low density for 6 h. The human lung cancer cell line H1299 and CL1-0 did not exhibit VM *in vitro*. However, the formation of VM was promoted by coculturing with CAFs, which were educated by cancer cells (**Figure 1B**). The effect of CAF on the facilitation of VM formation was also found for 12 h co-culture (**Supplementary Figure 1C**). Moreover, the results of the Matrigel plug analysis also indicated that CAFs increased VM formation in a mouse model (**Figures 1C, D**). These results indicated that CAFs contribute to VM formation in lung cancer.

**Table 1:** The characteristics of included lung cancer patients.

Case No.	VM formation	Age	Gender	Pathologic Findings	Stage (TNM)
1	No	46	F	Adenocarcinoma, grade 2	1A (T1N0M0)
2	No	50	F	Adenocarcinoma, grade 1	1A (T1aN0M0)
3	No	58	F	Adenocarcinoma, grade 2	1A (T1aN0M0)
4	No	63	M	Adenocarcinoma, grade 2	1B (T2aN0M0)
5	No	64	F	Adenocarcinoma, grade 2	1A (T1aN0M0)
6	No	48	M	Adenocarcinoma, grade 2	1A (T1aN0M0)
7	No	82	M	Adenocarcinoma, grade 2	4 (T1bN2M1b)
8	Yes	62	F	Adenocarcinoma, grade 3	4 (T1bN0M1b)
9	Yes	75	M	Adenocarcinoma, grade 2	2B (T3N0M0)
10	Yes	65	F	Adenocarcinoma, grade 3	3A (T2aN2M0)

### Notch Mediated Cell–Cell Contact of Cancer Cells and CAFs Contributes to VM Formation

**Figure 2:**
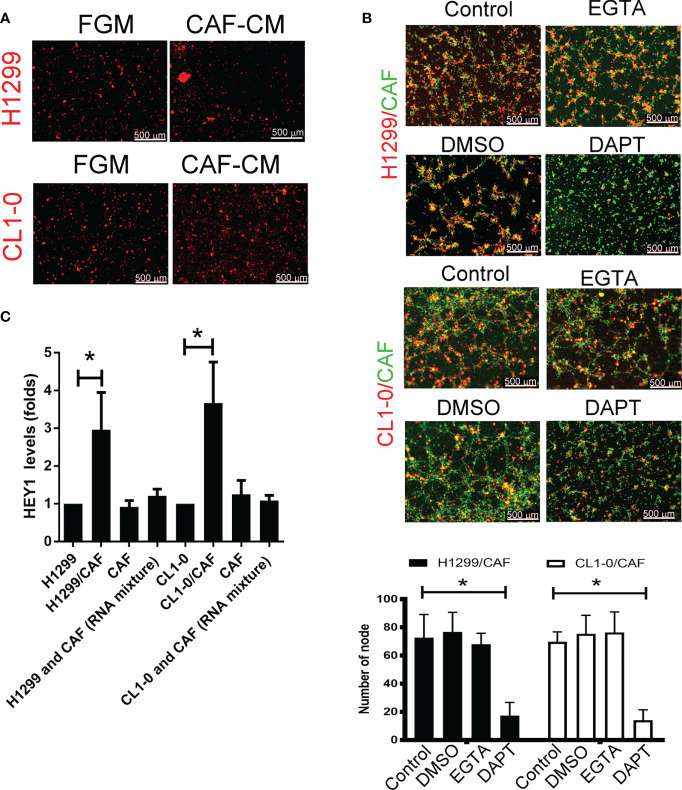
Cell–cell interaction contributes to CAF-derived vascular mimicry (VM) in lung cancer. **(A)** Cancer-associated fibroblasts (CAFs) condition medium (CM) failed to induce VM in H1299 and CL1-0 cells. The Fibroblast growth medium (FGM) or CAF’s CM was added on top of growth factor reduced Matrigel containing H1299 or CL1-0 cells for 6 (h) The VM structure was observed using a fluorescence microscope. **(B)** Disruption of Notch cell–cell contact prevented VM mediated by CAFs. CL1-0, H1299 cells, CAFs, or lung cancer cells mixed with CAFs were seeded on top of growth factor reduced Matrigel, and dimethyl sulfoxide (DMSO, vehicle control, 0.1%), Egtazic acid (EGTA, 2.5 mM), or γ-secretase inhibitors (DAPT, 2 μM) were added into the media. **(C)** Upregulated NICD target HEY1 gene expression in H1299 and CL1-0 cells cocultured with CAFs. The level of HEY1 was assessed by RT-qPCR. All results are representative of at least three independent experiments. Each value is the mean ± SD of three determinations; *P < 0.05.

To examine the type of cell–cell communications between cancer cells and CAFs that affect tumor growth, we assessed soluble factors by collecting the condition medium (CM) of CAFs. As shown in **Figure 2A**, the CAF supernatant did not stimulate H1299 or CL1-0 cells to format capillary-like structures (**Figure 2A**), suggesting that direct cell–cell contact, but not soluble factors, play a major role in cancer cell–CAF-consisting VM.

To examine which type of cell–cell contact affects VM formation, we used cadherin and Notch signaling inhibitors to assess VM formation. Although egtazic acid (EGTA) failed to interrupt VM formation, γ-secretase inhibitor DAPT reduced VM formation in H1299 or CL1-0 cells promoted by CAFs (**Figure 2B**), suggesting that Notch-mediated signaling contributes to VM formation. Moreover, the expression of NICD target HEY1 increased in both H1299 and CL1-0 when they were mixed with CAFs (**Figure 2C**).

### Notch2 and Jagged1-Mediated Signaling Is Involved in VM Formation

**Figure 3:**
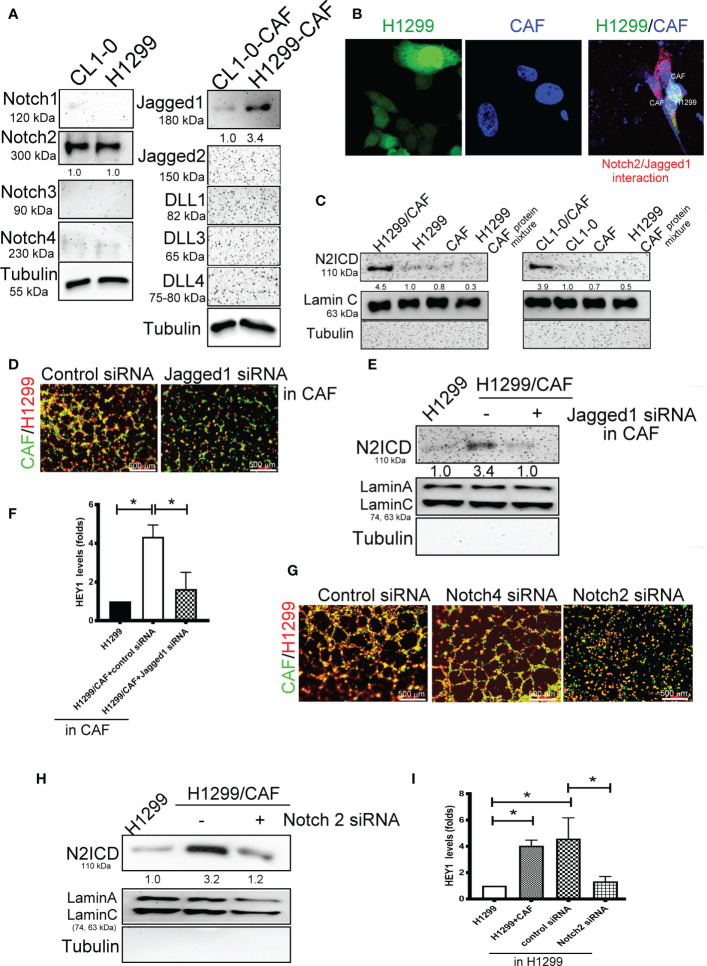
Interaction of Notch2 and Jagged1 between CAFs and lung cancer cells. **(A)** Expression of Notches and their ligands in lung cancer cells and their CAFs, respectively. **(B)** Close interaction of Nocth2 and Jagged1 between H1299 cells and CAFs as determined by proximity ligation assay (PLA). H1299-GFP cells, CAFs (BioTracker 400 Blue cytoplasmic membrane dye), and H1299-GFP cells mixed with CAFs were committed to PLA analysis (red). **(C)** Increased NOTCH 2 intracellular domain (N2ICD) nuclear translocation in H1299 cells. Cells were collected and the nuclear and cytosol fractions were extracted using a commercial kit. Equal levels of protein were assessed by immunoblotting. **(D)** Inhibition of Jagged1 in CAFs reduced the VM formation of H1299 cells. Knockdown of Jagged 1 in CAFs reduced **(E)** N2ICD nuclear translocation, and **(F)** HEY1 upregulation driven by CAFs in H1299 cells. Knockdown of Notch2 in H1299 reduced **(G)** VM formation, **(H)** N2ICD nuclear translocation, and **(I)** HEY1 upregulation driven by CAFs. CAFs and H1299 cells were transfected with a control, Jagged1 (for CAFs), or Notch2 (for H1299 cells) siRNA for 24 h, then CAFs were mixed with H1299 cells. The HEY1 mRNA and nuclear N2ICD levels were detected using a quantitative reverse transcription polymerase chain reaction and immunoblotting, respectively. All results are representative of at least three independent experiments. Each value is the mean ± SD of three determinations; *P < 0.05.

We further examined which Notch receptors and ligands are involved in VM formation in H1299 and CL1-0 cells in conjunction with CAFs. We assessed the expression of various Notch receptors in H1299 cells and Notch ligands in CAFs. High-level Notch2 and Jagged1 expression was detected in two lung cancer cell lines and their CAFs, respectively (**Figure 3A**). The results of a DuoLink^®^ proximity ligation assay (PLA), a technique detecting protein-protein interaction, also revealed a close interaction of Notch2 and Jagged1 when H1299 cells were mixed with CAFs (**Figure 3B**). In addition, the nuclear translocation of N2ICD, an active fragment of Notch2, was observed in H1299 and CL1-0 after they were cocultured with CAFs (**Figure 3C**). siRNAs targeting Notch2 and Jagged1 were used to silence Notch2 in H1299 cells and Jagged1 in CAFs. Notch2 and Jagged1 expression in H1299 cells and CAFs were succeeded knockdown by siRNA transfection at the protein level (**Supplementary Figures 2A, B**). Knockdown of Jagged1 in CAFs could significantly reduce VM formation, N2ICD nuclear translocation, and HEY1 upregulation in H1299 cells when H1299 cells were mixed with CAFs (**Figures 3D–F**). Similarly, silenced Notch2 in H1299 also inhibited VM formation (**Figure 3G**). Moreover, knockdown of Notch2 in H1299 cells also prevented N2ICD nuclear translocation (**Figure 3H**) and HEY1 upregulation (**Figure 3I**), which were induced by the H1299 cells and CAF coculture.

### Notch Mediated Cell–Cell Contact of Cancer Cells and CAFs Contributes to VM Formation

**Figure 4:**
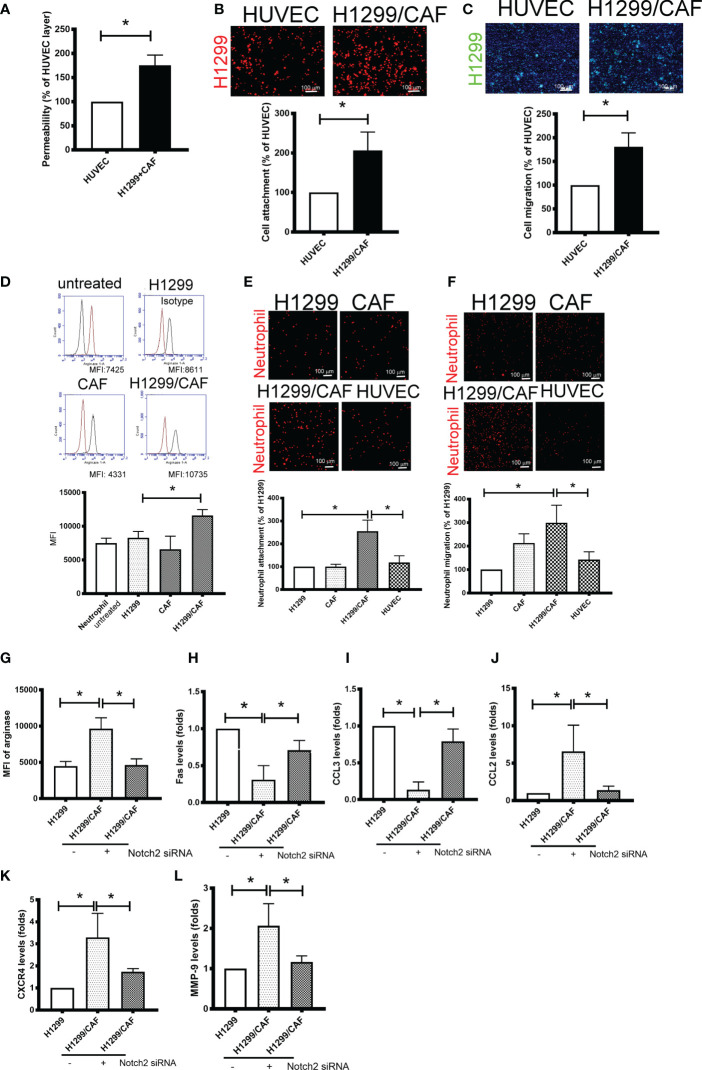
Vascular mimicry (VM) is a channel for cancer cells and neutrophil intravasation and extravasation. **(A)** VM Permeability. Human umbilical vein endothelial cells (HUVECs) or CAF/H1299 cells were seeded onto an insert (confluent monolayer in 1.0-μm-pore collagen-coated insert), and permeability was measured using FITC-dextran. **(B)** Cell adhesion and **(C)** transmembrane migration of H1299 cells in the VM network. **(D)** Arginase expression in neutrophil. **(E)** Cell adhesion and **(F)** transmembrane migration of neutrophil. Inhibition of Nocth2 by siRNA in H1299 prevented **(G)** arginase upregulation, **(H)** Fas and **(I)** CCL3 downregulation, and **(J)** CCL2, **(K)** CXCR4 and **(L)** MMP-9 enhancement in neutrophils. PHK67 or 26-stained H1299 cells or neutrophils were seeded on top of a confluent HUVEC or CAF/H1299 monolayer. Cell adhesion and transmembrane migration was assessed after 30 min and 24 h incubation, respectively. All results are representative of at least three independent experiments. Each value is the mean ± SD of three determinations; *P < 0.05. MFI, mean fluorescence intensity.

Because VM involves abnormal vessel-like structures, we hypothesized that cancer cells could intravasate into circulation through VM networks. To investigate the effect of VM on cancer metastasis, we also assessed cell adhesion, permeability, and transmembrane migration. Compared with the EC layer made by human umbilical vein ECs (HUVECs), higher permeability was found in VM (**Figure 4A**). In addition, the cell adhesion and transmembrane migration of H1299 cells were greater in VM vessels made by H1299 cells and CAFs than in the EC layer consisting of HUVECs (**Figures 4B, C**).

Because neutrophil infiltration and penetration is a critical characteristic of the metastatic niche (25), we evaluated the phenotypes, adherence, and transmembrane migration of neutrophils. As illustrated in **Figure 4D**, the supernatant collected from H1299 cells slight increased the population of N2-phenotype neutrophils (arginase+). The supernatant derived from H1299 cells mixed with CAFs further increased arginase levels in neutrophils. Consistent with changes in arginase expression, neutrophils treated with the supernatant harvested from H1299 cells mixed with CAFs produced significantly more N2-type neutrophils (Fas and CCL3 downregulation; CCL2 and CXCR4 upregulation) and tumor-promoting cytokines (MMP-9) than did untreated neutrophils or those treated with the supernatant from H1299 or CAF mRNA (**Supplementary Figures 3A–E**). In addition, VM induced by H1299 cells and CAFs not only increased neutrophil adhesion but also enhanced the transmembrane migration of neutrophils compared with the EC monolayer consisting of HUVECs (**Figures 4E, F**). The promotional effect of N2 polarization-induced VM created by H1299 cells and CAFs, including arginase upregulation and cytokine expressions, was inhibited when Notch2 expression was knocked down in H1299 cells by siRNA transfection (**Figures 4G–L**).

### Intercellular Adhesion Molecule-2 Contributes to VM-Mediated Neutrophil Infiltration

**Figure 5:**
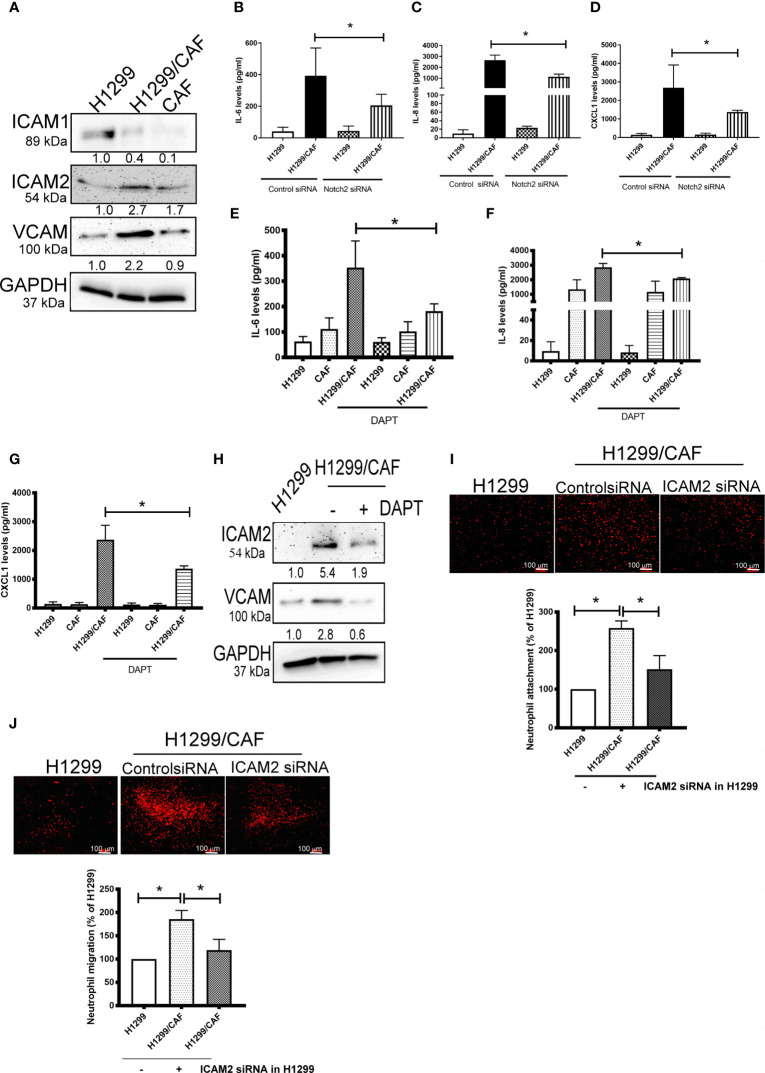
CAFs stimulate cytokine production of H1299 cells. **(A)** Expression of cell adhesion factors in H1299 cells. Protein levels of H1299 cells, CAFs, or H1299 plus CAF cells were assessed by immunoblotting. Knockdown of Notch2 in H1299 reduced the enhancement of **(B)** IL-6, **(C)** IL-8, and **(D)** CXCL1 in H1299 cells mixed with CAFs. γ-secretase inhibitors reduced the upregulation of **(E)** IL-6, **(F)** IL-8, and **(G)** CXCL1 in H1299 cells mixed with CAFs. H1299 cells, CAFs, or H1299 plus CAF cells were treated with or without DAPT for 24 h. Cytokine and intercellular adhesion molecule (ICAM) expression was assessed using a Luminex system or immunoblot, respectively. **(H)** ICAM2 and VCAM expression, **(I)** Cell adhesion and **(J)** transmembrane migration of neutrophils. PHK26-stained neutrophils were seeded on top of a confluent HUVEC or H1299/CAF monolayer. Cell adhesion and transmembrane migration were assessed after 30 min and 24 h of incubation, respectively. All results are representative of at least three independent experiments. Each value is the mean ± SD of three determinations; *P < 0.05.

To further test the specificity of Notch signaling in VM for neutrophil recruitment, we determined the expression of vascular cell adhesion protein (VCAM), intercellular adhesion molecule 1 and 2 (ICAM1 and 2), and several cytokines in VM that are chemoattractive agents for neutrophils. Immunoblot analysis demonstrated that the expression of ICAM2 and VCAM but not ICAM1 was greater in VM induced by H1299 cells and CAFs than in H1299 cells or CAFs alone (**Figure 5A**). Multiplex analysis also indicated that IL-6, IL-8, CCL8 and CXCL1 levels were increased in VM induced by H1299 cells and CAFs than in H1299 cells or CAFs alone (**Table 2**). The enhancement of these cytokines/chemokines (IL-6, IL-8, CXCL1) was prevented when Notch2 expression was knocked down in H1299 cells by siRNA transfection (**Figures 5B–D**). In addition, Notch signaling inhibition by DAPT prevented the upregulation of cytokines (IL-6, IL-8, CXCL1) and adherence molecules (ICAM2 and VCAM) in H1299 cells co-cultured with CAFs (**Figures 5E–H**). These results suggested that Notch signaling contributes to the upregulation of these cytokines, ICAM2 and VCAM in H1299 cells co-cultured with CAFs.

**Table 2:** The levels of cytokines in the H1299, CAF and H1299/CAF.

	H1299	CAF	H1299/CAF
IL-6	83.03 ± 19.02	111.48 ± 43.76	309.08 ± 123.07^*#^
IL-8	583.40 ± 9.21	1343.78 ± 65.73	2646.75 ± 497.44^*#^
CCL2	30.68 ± .8.92	61.83 ± 11.74	68.68 ± 34.34
CCL7	32.24 ± 2.76	39.98 ± 9.60	56.68 ± 12.99^*^
CCL8	5.59 ± 0.16	7.07 ± 1.08	9.97 ± 1.54^*#^
CXCL1	142.46 ± 74.93	133.62 ± 58.77	2366.25 ± 1076.71^*#^
sICAM1	11163.38 ± 1144.55	13190.50 ± 674.11	14567.13 ± 1614.94^*^

*, vs H1299, #, vs. CA (*, # p<0.05).

Because ICAMs expression controls neutrophil adhesion and transcellular migration (26–28), we assessed whether neutrophil infiltration contributes to ICAM2 upregulation in VM. ICAM2 expression was knocked down by ICAM2 siRNA transfection in H1299 cells (**Supplementary Figure 4**). ICAM2 silencing inhibited neutrophil adherence and transmembrane migration in VM consisting of H1299 cells and CAFs (**Figures 5I, J**).

### Inhibition of VM Amends the Therapeutic Effect of the Anti-VEGF Antibody Against Lung Cancer

**Figure 6:**
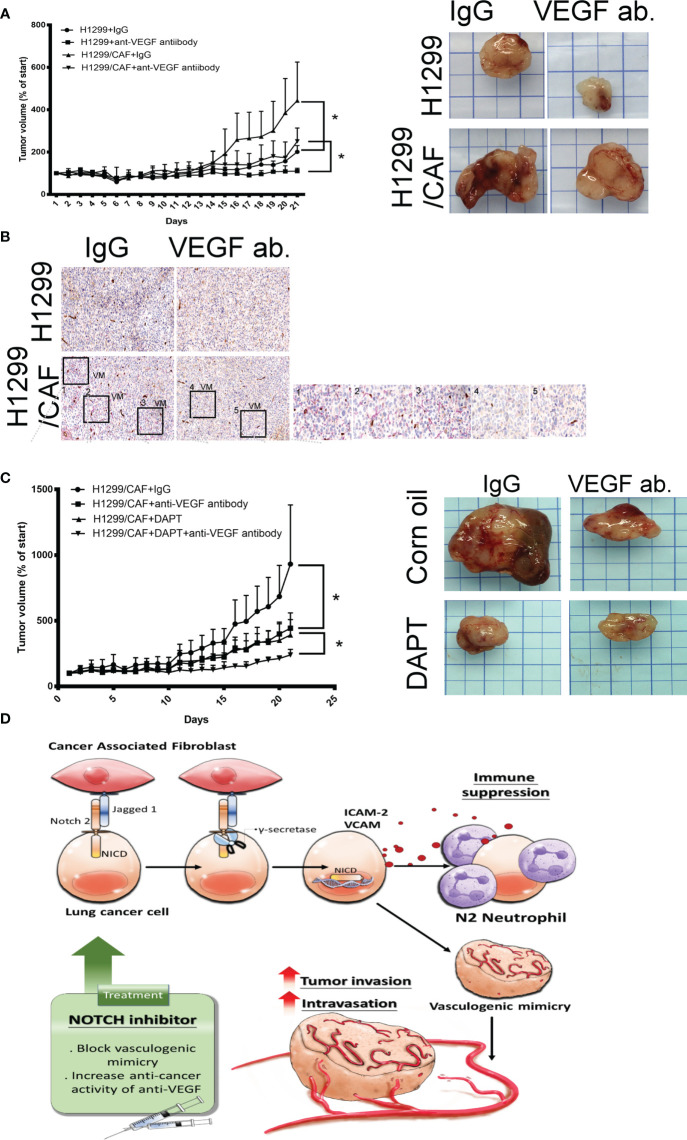
Inhibition of Notch signaling increases the therapeutic effect of anti-VEGF antibodies *in vivo*. **(A)** CAFs reduced the effect of anti-VEGF antibodies against lung cancer. **(B)** VM network in the tumors of mice. H1299 or CAF/H1299 cells were mixed with high-concentration Matrigel. Mice received either immunoglobulin G (IgG) or anti-VEGF antibodies (IP, 10 μg/mouse, 2 times/week). Matrigel plugs were harvested 21 days after subcutaneous injection into nude mice. **(C)** Notch inhibitor enhances anticancer activity of anti-VEGF antibodies *in vivo*. CAF/H1299 cells were mixed with Matrigel and subcutaneously injected into nude mice. Mice received IgG/vehicle control, IgG/DAPT, anti-VEGF antibody/vehicle, or anti-VEGF antibody/DAPT. Matrigel plugs were harvested 43 days (28-day treatment) after subcutaneous injection into nude mice. Tumor volume was measured every day with calipers. IgG or antibody treatment: IP, 10 μg/mouse, 2 times/week; vehicle or DAPT treatment: po, 5 mg/kg, 3 times/week. **(D)** Proposed model of CAF-mediated VM in lung cancer. Each value is the mean ± SEM; *P < 0.05.

A study indicated that VM plays a critical role in the chemoresistance of anti-VEGF antibodies therapy (7). Thus, we assessed whether CAFs impair the effect of anti-VEGF antibody in lung cancer. As illustrated in **Figure 6A**, CAFs increased H1299 lung cancer growth in mice. H1299 tumor growth was inhibited by anti-VEGF antibodies (IP, 10 μg/mouse, 2 times/week). However, the therapeutic effect of anti-VEGF antibodies was reduced because of CAF presence (**Figure 6A**). In addition, VM structures were observed in the tumor with CAFs (**Figure 6B**).

Next, we assessed whether inhibition of VM induced by lung cancer and CAFs with γ-secretase inhibitors amends the therapeutic efficacy of anti-angiogenesis in lung cancer. Both anti-VEGF antibodies (IP, 10 μg/mouse, 2 times/week) and DAPT (po, 5 mg/kg, 3 times/week, 4 weeks) alone inhibited lung cancer growth, although to different extents (**Figure 6C**). After 28 days of treatment, the mean tumor weight was 1.473 g in the control group. Anti-VEGF antibody treatment and DAPT were significantly effective in reducing the growth of tumor (0.884 and 0.85 g for anti-VEGF antibody and DAPT, respectively). A combination of anti-VEGF antibody treatment and DAPT further regressed tumor growth (0.428 g) in animal models, suggesting that Notch inhibition could reduce anti-VEGF antibody resistance due to VM in lung cancer (**Figure 6C**).

## Discussion

VM networks play a crucial role in promoting cancer metastasis and chemoresistance to angiostatic therapy (29, 30). The occurrence of malignancies with functional vascular-like networks is strongly correlated with advanced cancer aggressiveness and poor clinical outcomes in patients with cancer (30, 31). The VM formation consisted of capillary network, channel and tubular structure *in vitro* (32). The interaction occurring in the TME between cancer and CAFs not only effectively enhances the malignant features of cancer that allow tumorigenesis and proliferation but also confers an optimal niche for tumor cell advancement and metastasis (8, 33). This study indicated that CAFs are collaborators with lung cancer cells in VM formation by Notch2 and Jagged1 interaction. VM structures consisted of cancer cells, and CAFs contribute to increased cancer intravasation and neutrophil infiltration (**Figure 6D**). The crosstalk of cancer cells and CAFs by Notch2 and Jagged1 in the TME studied here confirms that γ-secretase inhibitors could be a promising strategy for improving anti-VEGF antibody treatment in lung cancer.

In contrast to Notch1 and Notch3, which are regarded as oncogenic factors responsible for lung cancer development, whether Notch2 has similar roles should be further investigated. Studies have demonstrated that Notch2 contributes to the increase of cancer stem cell properties and impairment of CD8+ T cell anticancer activity in patients with lung cancer (27, 34, 35). In addition, Notch2 expression is positively associated with cancer recurrence rate (36). Our results revealed that Notch2 participates in shaping the TME by interacting with CAFs to form VM networks. The loose junctions of VM networks allow the intravasation of cancer cells into circulation. In addition, Notch2 activation triggers ICAM2 upregulation, resulting in neutrophil recruitment. By contrast, inhibition of Notch2–Jagged1 interaction reduced VM formation and cancer growth *in vivo*, indicating that VM networks made by CAFs and cancer cells provide a beneficial TME for lung cancer.

Neutrophils are one of the most abundant immune cells that not only are a vital component of innate immunity but also contribute to adaptive immunity regulation by interacting with various adaptive immune cells (37, 38). The multifaceted roles of neutrophils in contributing to cancer mainly result from the secretion of different effector factors under different statuses. Two neutrophil phenotypes, N1 and N2, have been used to define neutrophil subtypes with tumor-suppressing or tumor-promoting properties due to their immunostimulating and immunosuppressive activity, respectively, in the TME (39, 40). The number of N2 neutrophils in the TME implies poor prognosis for patients with cancer. Neutrophils are mainly located in tumor margins in early stages, but they can easily infiltrate into central sites in late stages. In addition, N1 neutrophils present in an early-stage tumor then transform into a tumor-promoting N2 phenotype during tumor progression. Such phenotypic conversion is induced by factors derived from cancer or stroma cells in the TME. Our study indicated that VM networks consisting of cancer and CAFs increase N2 neutrophil polarization, which expresses high levels of arginase, CCL2, CXCR4 and MMP-9. By contrast, the anticancer factor Fas decreased in neutrophils if they were stimulated by VM structures. Moreover, the high permeability of VM allows neutrophil penetration, resulting in increased N2 neutrophil infiltration in cancer tissues. Therefore, we concluded that VM networks not only alter neutrophils from tumor-suppressing N1 to tumor-promoting N2 but also provide a critical channel for neutrophil infiltration.

Antiangiogenic therapy is a promising strategy for treating solid cancers; however, ineffectiveness and resistance are common problems in clinical settings (41). VM plays a crucial role in the development of antiangiogenic therapy resistance (42). Our results indicated that CAFs reduce the anticancer activity of anti-VEGF antibodies, whereas inhibiting CAF–cancer cell interaction potentiates the effect of antiangiogenic antibodies in a mouse model. In conclusion, the results of this study indicate the involvement of CAF–cancer cell interaction in the formation of VM structures and mediation of anti-VEGF antibody resistance in lung cancer. γ-secretase inhibitors can successfully block Notch2–Jagged1 interaction to prevent VM formation, thus providing new avenues for using this strategy as adjuvants to improve the efficiency or resistance of antiangiogenic therapies for lung cancer. Overall, our study demonstrated that cell–cell contact between CAFs and cancer cells prime successful VM formation and weakens intracellular junctions to support cancer growth and intravasation as well as shapes an immunosuppressive niche TME.

## Conclusions

Our results elucidate a new mechanism by which CAFs facilitate VM formation and permeability through a previously undescribed Notch2–Jagged1 interaction. This cell–cell communication by direct cell–cell contact, which connects CAFs and cancer cells, may offer innovative alternatives for preventive or adjuvant therapies for lung cancer patients.

## Data Availability Statement

The original contributions presented in the study are included in the article/**Supplementary Files**, further inquiries can be directed to the corresponding author.

## Ethics Statement

The studies involving human participants were reviewed and approved by the Institutional Review Board of Kaohsiung Medical University Hospital. The patients/participants provided their written informed consent to participate in this study. The animal study was reviewed and approved by Institutional Animal Care and Use Committee of Kaohsiung Medical University.

## Author Contributions

Conceptualization, YMT and YLH; methodology, YCH, CYC, PHT and SHL; validation, WAC and YLH; formal analysis, JYH and YLH; data curation, YCH and YLH; resource, YLH; writing—original draft preparation, YMT and KLW; visualization, YMT; supervision, YLH. All authors contributed to the article and approved the submitted version.

## Funding

This study was supported by grants from the Ministry of Science and Technology (Grant No. MOST 110-2314-B-037 -124 -MY3, 109-2314-B-037-091, 109-2314-B-037-126-MY2, 108-2320-B-037-024-MY3, 108-2314-B-037-094-, 108-2314-B-037-095- and 107-2314-B-037-107-MY3), Kaohsiung Medical University (Grant No. KMU-DK 109002, KMU-DK108003, KMU-DK108008, KMU-TC108A03-5) and Kaohsiung Medical University Hospital (KMUH-109-9R12 and KMUH-109-9M13).

## Conflict of Interest

The authors declare that the research was conducted in the absence of any commercial or financial relationships that could be construed as a potential conflict of interest.

## Publisher’s Note

All claims expressed in this article are solely those of the authors and do not necessarily represent those of their affiliated organizations, or those of the publisher, the editors and the reviewers. Any product that may be evaluated in this article, or claim that may be made by its manufacturer, is not guaranteed or endorsed by the publisher.
