# Supplementary figures and images for: Cooperation Between Cancer and Fibroblasts in Vascular Mimicry and N2-Type Neutrophil Recruitment via Notch2–Jagged1 Interaction in Lung Cancer
Source: Front Oncol. 2021 Aug 17;11:696931. doi: 10.3389/fonc.2021.696931 (PMC8415962; doi:10.3389/fonc.2021.696931)

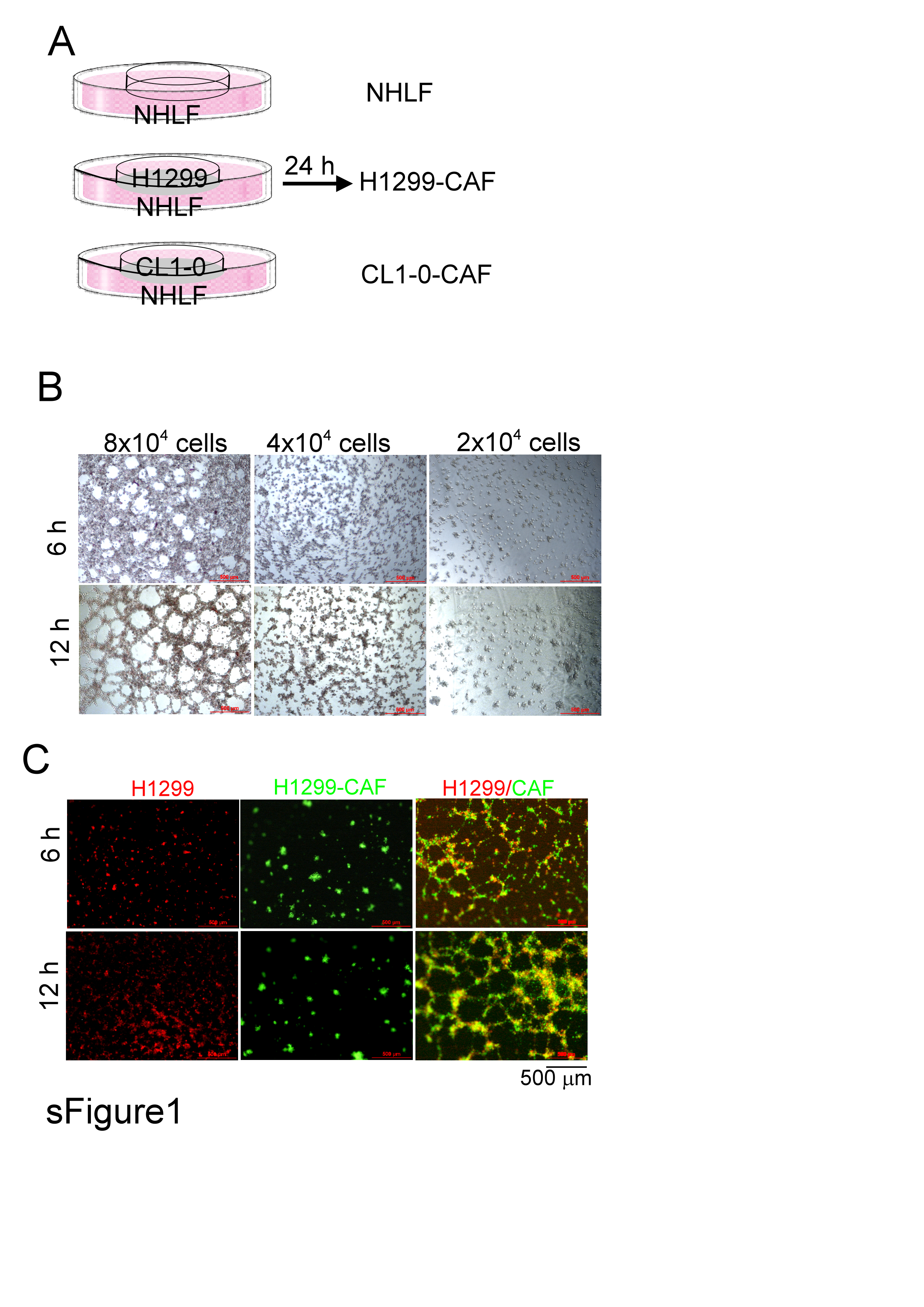

Supplement: Supplementary file 1 [file Image_1.tif]

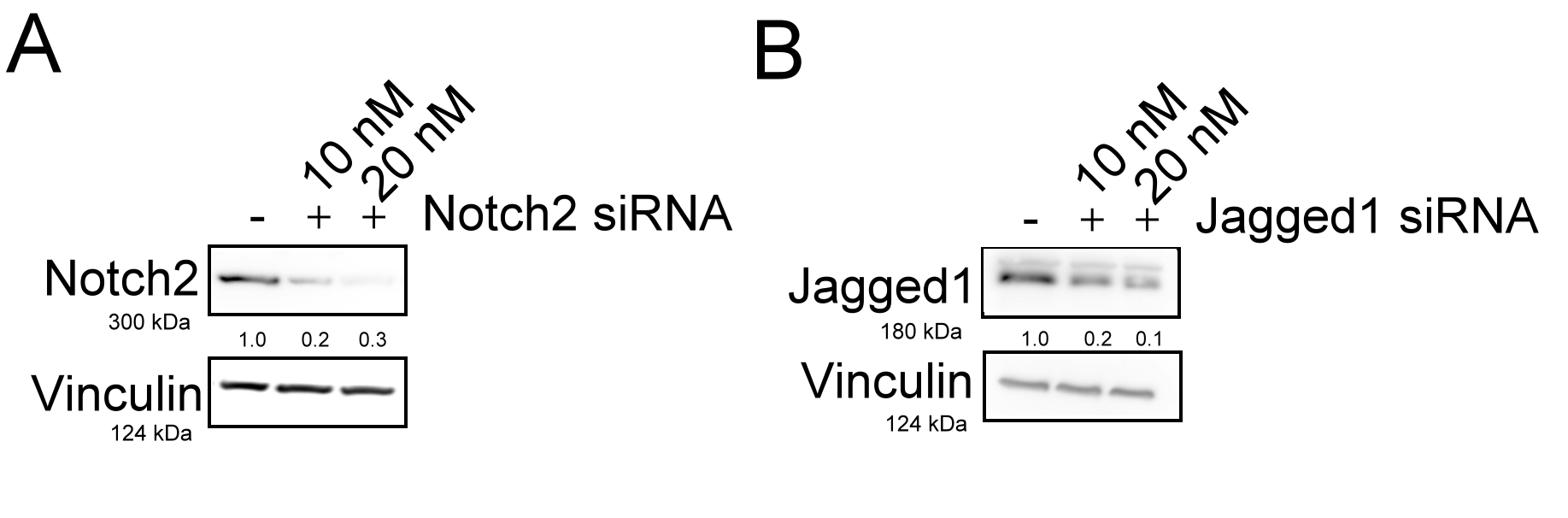

Supplement: Supplementary file 2 [file Image_2.tif]

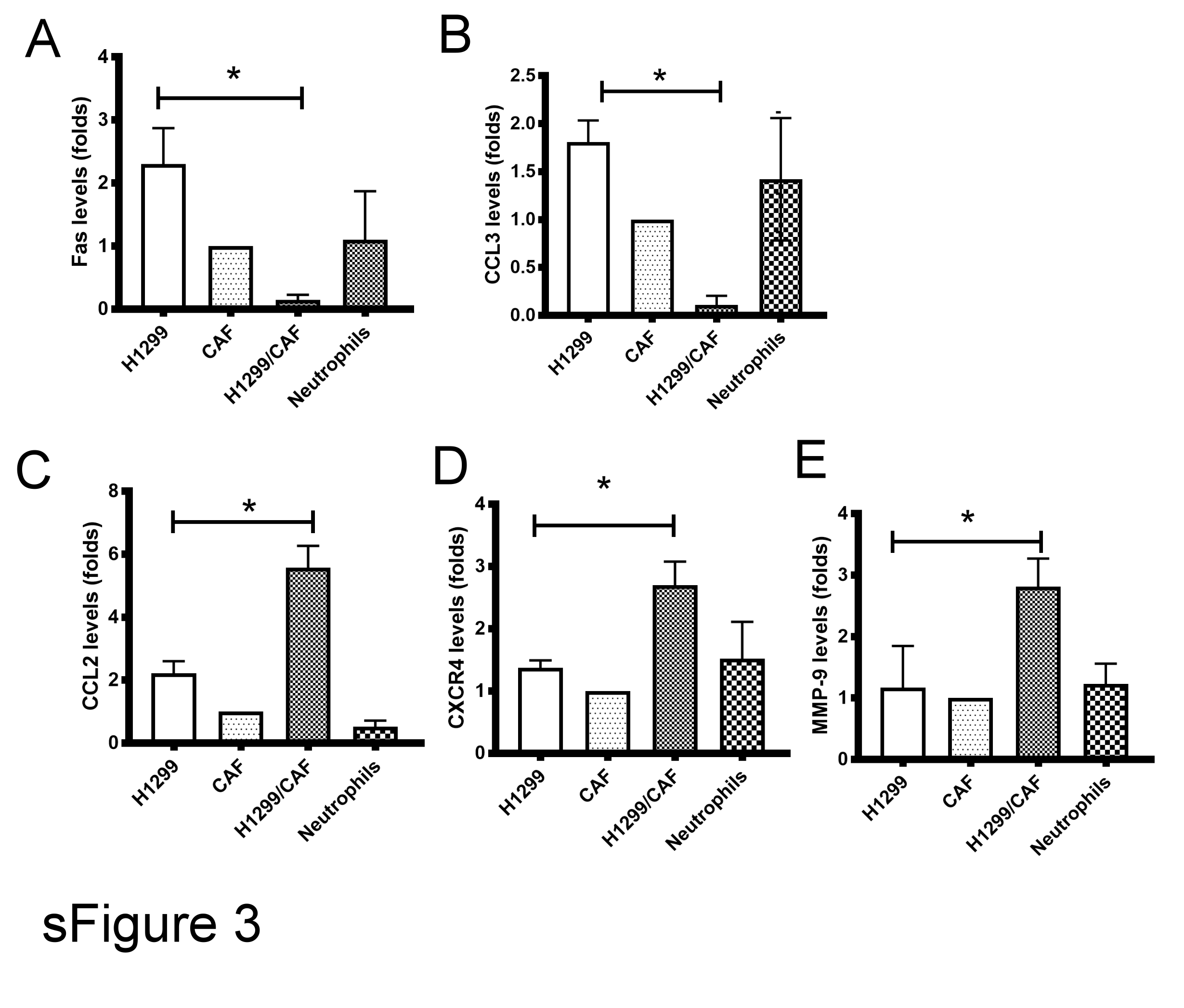

Supplement: Supplementary file 3 [file Image_3.tif]

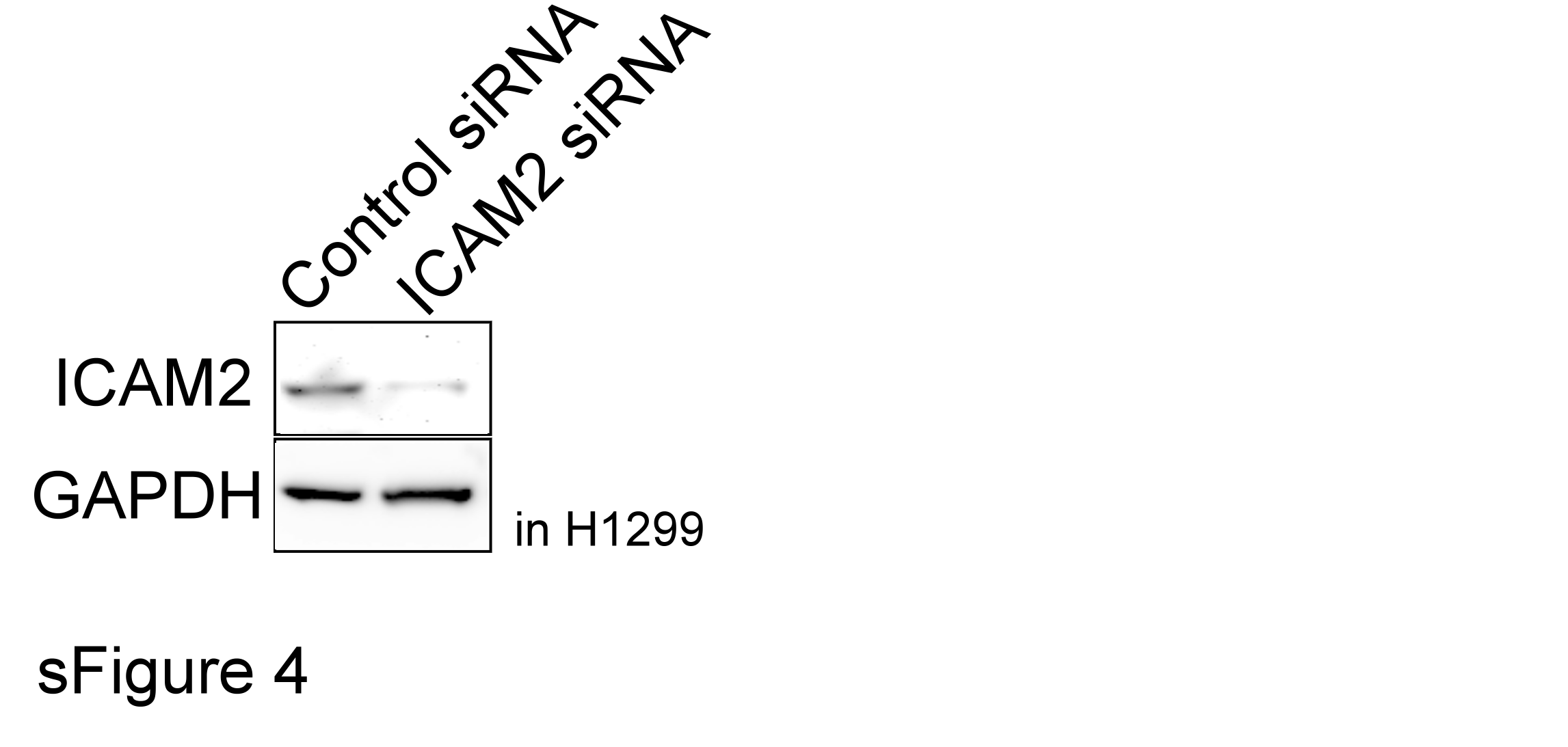

Supplement: Supplementary file 4 [file Image_4.tif]
