# Supplementary material for: Cooperation Between Cancer and Fibroblasts in Vascular Mimicry and N2-Type Neutrophil Recruitment via Notch2–Jagged1 Interaction in Lung Cancer
Source: Front Oncol. 2021 Aug 17;11:696931. doi: 10.3389/fonc.2021.696931 (PMC8415962; doi:10.3389/fonc.2021.696931)
Supplement: Supplementary file 5 [file Table_1.docx]

**sTable 1. The sequence of primers**

| Gene | Primer sequence |
| --- | --- |
| Fas | left:  ggaccctcctacctctggtt;  right: GAGGACAGGGCTTATGGCAG |
| CCL2 | left:  agccaccttcattccccaag;  right: CTCCTTGGCCACAATGGTCT |
| CCL3 | left:  catcacttgctgctgacacg;  right: TTCTGGACCCACTCCTCACT |
| CXCR4 | left:  atcagtctggaccgctacct;  right: ATCTGCCTCACTGACGTTGG |
| MMP-9 | left:  acctcgaactttgacagcga;  right: ATGCCATTCACGTCGTCCTT |
| HEY1 | left:  agttgcgcgttatctgagca  right: CGATGTGCGGGTGATGTCC |
| GAPDH | left:  GAGTCAACGGATTTGGTCGT;  right: TTGATTTTGGAGGGATCTCG |
